# Spot Tracking and TDC Sharing in SPAD Arrays for TOF LiDAR

**DOI:** 10.3390/s21092936

**Published:** 2021-04-22

**Authors:** Vincenzo Sesta, Fabio Severini, Federica Villa, Rudi Lussana, Franco Zappa, Ken Nakamuro, Yuki Matsui

**Affiliations:** 1 Politecnico di Milano, Dipartimento di Elettronica, Informazione e Bioingegneria, Piazza Leonardo da Vinci 32, I-20133 Milano, Italy; vincenzo.sesta@polimi.it (V.S.); fabio.severini@polimi.it (F.S.); federica.villa@polimi.it (F.V.); rudi.lussana@polimi.it (R.L.); 2 OMRON Corporation, Technology and Intellectual Property H.Q., 9-1 Kizugawadai, Kizugawa-City, Kansai Science City, Kyoto 619-0283, Japan; ken.nakamuro@omron.com (K.N.); yuki.matsui@omron.com (Y.M.)

**Keywords:** Single Photon Avalanche Diode (SPAD), Light Detection and Ranging (Lidar), Time-of-Flight (TOF) measurements, Region of Interest (ROI), Time-to-Digital Converter (TDC)

## Abstract

Light Detection and Ranging (LiDAR) is a widespread technique for 3D ranging and has widespread use in most automated systems that must interact with the external environment, for instance in industrial and security applications. In this work, we study a novel architecture for Single Photon Avalanche Diode (SPAD) arrays suitable for handheld single point rangefinders, which is aimed at the identification of the objects’ position in the presence of strong ambient background illumination. The system will be developed for an industrial environment, and the array targets a distance range of about 1 m and a precision of few centimeters. Since the laser spot illuminates only a small portion of the array, while all pixels are exposed to background illumination, we propose and validate through Monte Carlo simulations a novel architecture for the identification of the pixels illuminated by the laser spot to perform an adaptive laser spot tracking and a smart sharing of the timing electronics, thus significantly improving the accuracy of the distance measurement. Such a novel architecture represents a robust and effective approach to develop SPAD arrays for industrial applications with extremely high background illumination.

## 1. Introduction

Light Detection and Ranging (LiDAR) is a technique for evaluating distances, consisting of measuring the round-trip time of an optical signal emitted by a light source (typically a pulsed laser), backscattered by the target and received by a suitable detector [1,2,3,4]. Single Photon Avalanche Diodes (SPADs) and SPADs arrays have been successfully exploited in a broad variety of LiDAR applications, such as surveillance, security, and autonomous vehicles [5,6,7,8,9,10,11,12,13,14,15,16,17,18,19], taking advantage of their single-photon sensitivity, ultra-precise time-tagging capability, and high frame rates. The unavoidable interaction with the external environment and with a strong background illumination are the major challenges in high-performing LiDAR sensors, particularly in industrial environments, such as in the case analyzed in this paper. Different approaches can be implemented to estimate objects’ distance through optical measurement of back-reflected photons. The most suitable techniques are based on *photon-timing, time-gated photon-counting,* or *multi-photon* approaches, all discussed herein.

**Figure 1:**
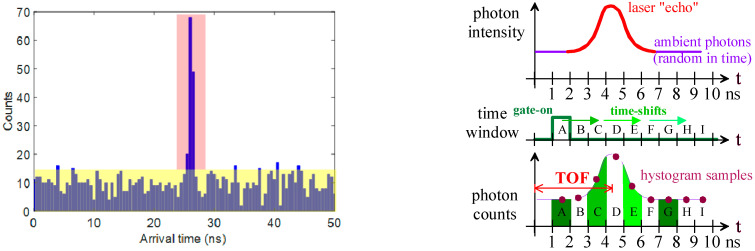
(**Left**) Typical photon-timing mode measurement: randomly scattered events (yellow background) are due to ambient photons (and dark count rate), while time-correlated photons close to each other (red area) belong to the laser pulse return “echo”. (**Right**) Typical time-gated photon-counting procedure: a gate-on window, in which the SPAD is activated, is progressively shifted across the entire full-scale range, thus reconstructing the optical “echo” of the laser pulse.

In *photon-timing mode*, the time delay between the arrival time of every incoming photon and the laser pulse excitation is recorded and accumulated into a histogram, as exemplified in Figure 1 (left). As shown by the yellow area, events due to ambient photons (not correlated to the laser pulse) and to the SPAD intrinsic noise (the dark count rate, DCR) are randomly scattered across the time axis. Instead, photons belonging to the optical “echo” of the laser pulse, traveling back-and-forth from the object in the scene, hit the SPAD detector at a specific time-delay, corresponding to the time-of-flight (TOF) of the laser photons (red area). The object distance is computed from the centroid of the TOF peak in the histogram (better if limited to just the bins corresponding to the peak).

This approach requires a low time-jitter SPAD and time-measuring device, usually a Time-to-Digital Converter (TDC) with sub-ns timing resolution and jitter. The main drawback of the photon-timing approach is the large area occupation of the timing electronics, which is usually much larger than the SPAD active area, thus translating into a very low fill factor (FF), likely of few percent at the most, and consequently a high photon loss.

Circuit complexity can be reduced by employing the so-called *time-gated photon-counting mode,* in which incoming photons are counted and recorded through digital counters, which are way simpler than TDCs and require a much smaller area, eventually yielding to higher fill-factor arrays [20]. Figure 1 (right) exemplifies a typical time-gated photon-counting measurement where a gate time-window shifts across the full-scale range and photons are simply counted therein after many laser pulse repetitions, so to finally identify the laser “echo” where the signal photon return is higher. Gate-on duration is typically in the order of few nanoseconds, the delay step is of some tens of picoseconds, and the maximum measurable range is a few tens of meters. The disadvantage of this approach is that photons back-reflected from the target with a TOF outside the gate-on time-windows are lost, since they cannot be detected. This low operating duty-cycle causes a high photon loss, thus impairing distance measurement precision, as it will be clarified in Section 2. For this reason, this approach will not be considered in this study.

*Multi-photon mode* is similar to photon-timing mode with single SPADs but applied to an analog Silicon Photo Multiplier (SiPM), which is a very compact array of independent SPADs, with a series of self-quenching resistors each, all connected in parallel so to act as a single ensemble detector. In this approach, single-shot measurements are possible, because the SiPM detector is sensitive to multi-photons hitting the active area either in different time instants or even concurrently. Having just an anode and a cathode, the ensemble output current can be sensed by means of a Trans-Impedance Amplifier (TIA), so to provide an output voltage proportional to the number of concurrently hitting photons. As an alternative, digital SiPMs include an Active Quenching Circuit (AQC) connected to each SPAD; all of an AQC’s digital outputs are combined together into a single digital output by means of an OR logic gate. Typically, the overall FF of a SiPM is very high, even higher than 70%, as an SPAD’s sensitive areas are very close to each other, while auxiliary components (i.e., self-quenching resistors or AQCs) have an optimized layout for low area occupation. *Multi-photon mode* can be herein exploited, since a very strong laser pulse can concurrently trigger many SPADs, thus producing an output current pulse whose peak amplitude is proportional to the number of detected photons. Conversely, dark counts and background photons that are not correlated in time only contribute as randomly scattered pulses with low intensity, since they seldom pile up concurrently one on top of the other. A smart exploitation of multi-photon detectors such as SiPMs may consist in employing an analog discriminator to threshold the output current, at a sufficiently high value to avoid ignition due to randomly scattered background events, while being reasonably low to be triggered by concurrent photons belonging to the laser pulse excitation. With respect to the standard photon-timing mode, in which each photon detection translates into a TDC conversion, the conversion is triggered only one every N_THRESHOLD_ photons, resulting in a precision worsened by the factor √N_THRESHOLD_, as it will be clarified in Section 2. 

Table 1 lists some SPAD imagers for TOF-LiDAR reported in literature; as it can be noted, the number of pixels can vary from just a few tens up to many thousands. Multi-photon arrays are organized in pixels containing up to hundreds of SPADs. High fill- factor values (above 25%) can be easily achieved with both multi-photon arrays and photon counting arrays but usually not in photon timing mode. For improving ranging performance (i.e., resolution and range) in photon-timing and time-gated photon counting arrays, the fill factor decreases due to the larger area required for the in-pixel electronics. Instead, in multi-photon arrays, the high fill-factor is obtained, since the TDCs are shared among SPADs, optimizing the power consumption and the occupation area. In photon-timing and multi-photon SPAD arrays, resolution and range correspond to the TDC resolution and full-scale-range, respectively (through half the speed of light, c/2); instead, in photon counting arrays, they respectively correspond to the minimum gate shift and to the gate shift range. Recently, 3D-stacked SPAD imagers for TOF-LiDAR have been proposed in the literature [18,19], employing backside-illuminated SPADs on the top tier and control/processing/readout electronics on the bottom tier.

Moreover, commercial TOF ranging sensors are available on the market for instance from STMicroelectronics, AMS, Sharp, and Bosch. Usually, they reach up to 2.5 m range and are sold as a system-in-package solution with illuminator, silicon SPAD array chip and housing, as a single-point TOF ranging arrays [21,22,23].

## 2. Specifications and Performance Requirements

After introducing the main approaches for measuring distances through optical techniques, this section will drill down on the project specifications and required performance for obtaining a TOF-based sensor, to be included into a compact single-point rangefinder that must ensure optimal performance even in the presence of a strong background illumination. The first part of the discussion will eventually provide an estimate of the minimum signal photons to be detected, the signal detection rate, and the background detection rate, all of which will be of primary interest in the identification of the optimal sensor architecture, as discussed in the following sections.

The requirements are summarized in Table 2. As the sensor is expected to be employed in an automated assembly line, a range of approximately 1 m suffices, corresponding to a 6.7 ns TOF. Then, given the 3 ns maximum pulse-width of the emitted laser pulse, the final Full-Scale Range (FSR) of the timing electronics should be the sum of the two. These specifications, along with the required response time and the given laser pulse repetition rate, will be used in Section 3.3 for properly sizing the TDC electronics.

Note that a wider laser pulse is preferred, since it represents a typical constraint set by cost reasons in real applications where many detectors are required. A narrower pulse width can be considered but a 3 ns laser pulse is taking into account to consider the worst-case scenario.

The TOF measurement precision (±3 σ), limited to ±10 mm, must be ensured even in the presence of a very strong background illumination, estimated to be 3000 lx(corresponding to a typical neon halogen lamp) and must apply also to black-colored objects (i.e., with 3% reflectivity). Note that we considered only the halogen lamp as illumination, since it is employed in typical industrial environment as specified in the project requirements. In general, by neglecting any background contribution and considering a Gaussian laser pulse with σ_single−shot_ width, and neglecting any contribution from electronics jitter and quantization, the best achievable TOF precision (of the photon-timing histogram centroid) σ_TOF_ is given by:

(1) $$\sigma_{\mathrm{TOF}}=\frac{\sigma_{\sin\mathrm{gle}-\mathrm{shot}}}{\sqrt{N_{\mathrm{photons}}}}.$$

Hence, the higher the number of collected events N_photons_ within the peak of the TOF distribution, the more precise the estimation of the distance based on the centroid computation. Thus, in order to achieve the desired 10 mm three-sigma precision (i.e., σ_TOF_ = 22 ps), starting from the 3 ns FWHM (i.e., σ_single-shot_ = σ_laser_ = 1.27 ns) laser pulse width, 3300 photon detections are required over the 500 laser repetitions; i.e., about 7 photons have to be detected per each laser shot.

However, the achievable precision is not only related to the sole laser pulse width, but to the SPAD and to the TDC time-jitter as well, hence:

(2) $$\sigma_{\sin\mathrm{gle}-\mathrm{shot}}=\sqrt{\sigma_{\mathrm{laser}}^{2}+\sigma_{\mathrm{SPAD}}^{2}+\sigma_{\mathrm{TDC}}^{2}}$$

where σ_TDC_ includes both the contribution of TDC jitter (σ_jitter_) and of quantization noise (σ_q_). By considering a very precautionary value of 500 ps for the SPAD and TDC contributions (σ_SPAD_ and σ_TDC_), which should be quadratically added to the σ_laser_ = 1.27 ns laser pulse width, the total number of required detections increases to about 3800, i.e., to about 7.6 detections on average per laser pulse.

Since the total σ_TOF_ uncertainty is proportional to √N_photons_, any source of photon loss (e.g., fill factor, detection efficiency, optical losses) directly impairs the measurement precision. Moreover, since more than one photon detection is required per each laser pulse, one single SPAD detector is not enough: the reason being that the SPAD features a typical dead time in the order of tens of nanoseconds; i.e., the SPAD is kept off for a time duration that is longer than the 3 ns laser pulse, thus allowing only one photon detection per SPAD per pulse at most. Therefore, the overall detector must exploit the parallelization of several SPADs due to this “single-hit” limitation. Finally, all these considerations still neglect the detrimental effects of background illumination, which introduce a further source of uncertainty and might make it hard to achieve the aimed precision.

The expected *signal detection rate* can be evaluated by considering a Gaussian laser pulse with 100 mW peak power (ideally matched with the detection field of view) and the worst-case 3 ns FWHM pulse duration. Thus, the overall number of photons emitted by a single laser pulse at the wavelength of interest (i.e., λ = 660 nm) is about 10^9^ photons. Then, assuming an ideal Lambertian reflector with 3% reflectivity, just about 3·10^7^ photons will be back reflected, and just about 600 photons are accepted by the lens, taking into account the 10.7 mm lens diameter and 85% filter transmissivity and 85% lens transmissivity. Then, considering a 150 µm laser spot diameter imaged on the detector, the expected signal photon density is about 3.4 × 10^4^ photons/mm^2^ (per laser pulse).

**Figure 2:**
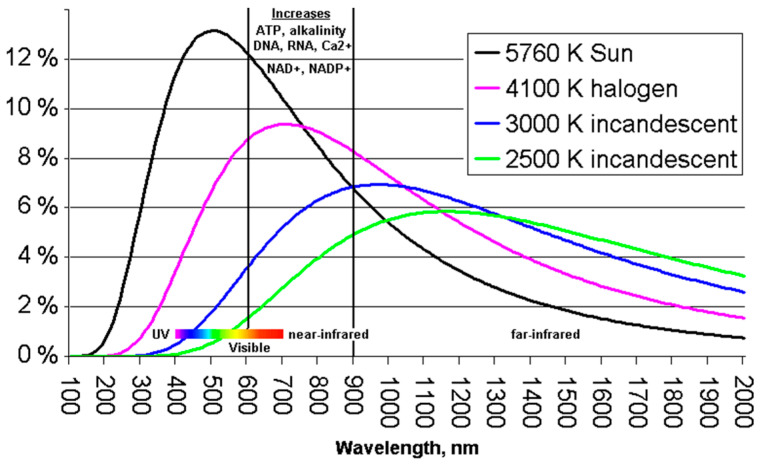
Typical spectral density of the light produced by a halogen lamp.

On the other hand, the expected *background detection rate* can be estimated starting from the 3000-lx background illumination of a halogen lamp, which translates in 39.1 W/m^2^, about 4% of which (i.e., 1.56 W/m^2^) is around the 660 nm transmission band (with 45 nm band pass filter width), as shown in Figure 2. By scaling it by the 50% reflectivity, 85% filter transmissivity, and 85% lens transmissivity, we achieve about 0.6 W/m^2^, or about 2 × 10^12^ ph/s/mm^2^ impinging on the SPAD array, at the 660 nm wavelength of interest. The minimum number of photons to be detected and the expected signal and background photon rates are summarized in Table 3.

## 3. Required Detector Performance

In this section, we compute the required detector performance to meet the project specification listed in Table 2. We illustrate the geometry chosen for the array and the required dimension of SPAD detectors. Furthermore, we analyze the SPAD and TDC performance to guarantee the required accuracy of TOF measurements.

A first parameter that can be defined is the maximum number of TOF samples based on the desired frame time and the laser pulse period, as given by:

(3) $$\mathrm{sample}\;\mathrm{number}=\frac{\mathrm{frame}\;\mathrm{time}}{\mathrm{emission}\;\mathrm{pulse}\;\mathrm{period}}=\frac{0.5\;\mathrm{ms}}{1\;µs}=500$$

### 3.1. Geometrical Considerations

**Figure 3:**
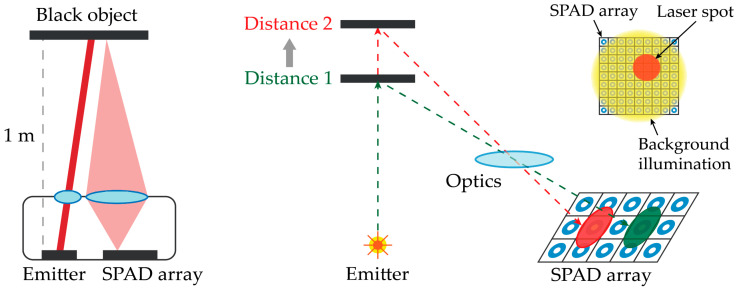
Optical setup with SPAD array and laser emitter (**left**) and the effect of the object variable distance on the actual position of the laser spot on the detector (**right**).

A typical optical setup of the array chip and the laser diode emitter is shown in Figure 3 (left). Due to the relative tilt between illumination spot and detection field of view, as the target distance changes, the laser spot experiences a unidimensional drift across the detector array; thus, it is imaged on different array positions, as depicted in Figure 3 (right). Therefore, a rectangular sensing area shape must be considered to better accommodate the spot shift along one dimension [16,17], while keeping the other one as short as possible in order not to increase the sensitive area to unwelcome ambient light. Given the 150 µm diameter of the laser spot on the SPAD detector array, the vertical dimension of the detector array should provide some more margin to compensate for the possible misalignment between sensing area and optics. Once the shape of the SPAD array area has been chosen, the size of the SPAD can be properly dimensioned as well as the number of SPADs to lay out in rows-by-columns across the overall SPAD array.

Let us consider an SPAD with a typical PDP (Photon-Detection Probability) of 20% at the wavelength of interest (660 nm) [24] and the signal photon density of 3.4 × 10^4^ ph/mm^2^ per laser pulse and the ambient photon density of 2 × 10^12^ ph/s/mm^2^, as computed in Section 2. In order to have only one signal photon hitting one SPAD per laser pulse at the most, the maximum SPAD area should be:

(4) $$\mathrm{SPAD}\;\mathrm{area}=\frac{1\;\mathrm{photon}}{3.4\times 10^{4}\frac{\mathrm{ph}}{{\mathrm{mm}}^{2}}\cdot 20\%}\approx 150{\;µm}^{2}$$

With a round-shaped active area SPAD, we obtain a diameter of about 14 µm: there is no advantage in using an SPAD with a diameter larger than 14 µm, while it could be possible to use smaller ones. Although SPADs with diameters narrower than 10 µm have been reported [25,26,27], they could suffer by a reduced actual PDP, due to edge effects and reduced fill-factor. With a 14 µm SPAD, the background photon rate is:

(5) $$\mathrm{Background}\;\mathrm{ph}\;\mathrm{rate}=2\times 10^{12}\frac{\mathrm{ph}}{s\cdot {\mathrm{mm}}^{2}}\times 20\%\cdot 10\mathrm{ns}\times 150{µm}^{2}=0.6\frac{\mathrm{ph}}{\mathrm{pulse}}$$

which is not negligible. Therefore, a proper sizing of the array should aim at maximizing the number of SPADs within the laser spot, hence minimizing the SPAD size (always <14 µm) while maximizing spot size and the array’s fill-factor.

### 3.2. SPAD Performance

**Figure 4:**
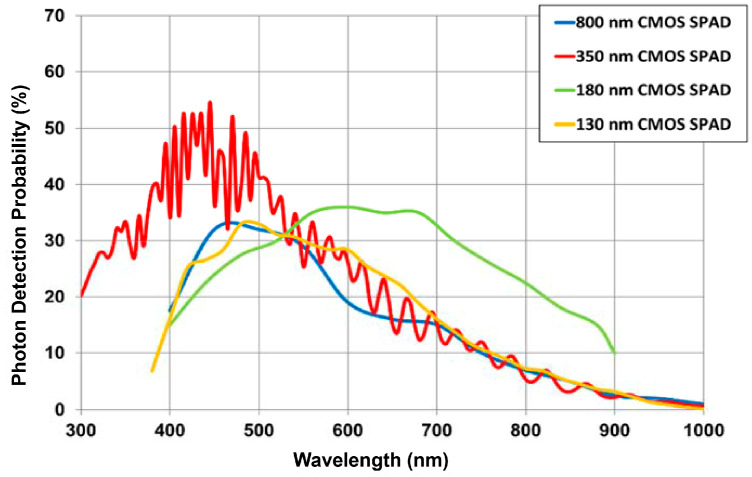
Photon detection probability of SPADs fabricated in different technologies (from [24]).

The performance of SPADs reported in the literature [5,8,11,12,13,14,15,16,17] demonstrates that they represent a valid candidate to develop a single-point Lidar TOF rangefinder. One of the most important parameters for the TOF Lidar array is the PDP at the operating wavelength. Figure 4 shows a typical PDP of the SPADs developed in different technology nodes [24]: PDP reaches good performance in CMOS technologies, which allow the integration of the detector and the front-end electronics in a monolithic device. Since the signal and the background photon rate are very high, low PDP is not critical and allows to avoid the count-rate saturation even with diameter higher than 8 µm. A higher PDP would involve using SPADs with diameter lower than 8 µm which in non-scaled technology nodes starts having issues in terms of Dark Count Rate (DCR) and side effects. The DCR is not critical, since it is in the order of a few thousand counts per second (cps) at most, which is definitely negligible when compared to the several megaphotons/s detected by each SPAD because of very high expected ambient background illumination [5,28,29]. Since the total time jitter is dominated by the wide 3 ns laser FWHM, also the SPAD timing jitter is not so relevant, being usually better than 100 ps [28,29].

Another critical performance for this application is the photon detection rate, which should be limited so to reduce the afterpulsing phenomenon [30], which causes spurious ignitions of the SPAD when re-armed after a triggering, due to carriers trapped during the previous avalanche current process that are then released. For this reason, the SPAD must experience a not nil dead-time, so to avoid most of the trapped carriers release and to reduce such spurious triggering: therefore, the front-end circuit should set a dead time of about 10 ns or longer.

### 3.3. TDC Performance

Since the single-shot precision is dominated by the 3 ns laser FWHM (variance σ = 1.27 ns), the Least-Significant Bit (LSB) of the TDC must be set shorter in order to make its σ_TDC_ uncertainty contribution negligible with respect to the laser variance σ_laser_ (see Equation (2). An LSB of about 100 ps or slightly shorter can be easily implemented in cost-effective CMOS technology [12,31], so to possibly allow for the exploitation of even better (shorter pulse) and more expensive lasers.

The FSR of the TDC is set by the maximum distance range to be detected, which is at least 1 m in our application (i.e., a TOF of 6.7 ns); thus, 10 ns FSR is a reasonable value, with a corresponding low number of bits (6–7 bits, given the 10 ns FSR and about 100 ps LSB), so a coarse TDC suffice, with no need to implement a finer TDC architecture [12,31].

Differential Non-Linearity (DNL) is not critical in TOF applications, since the distance estimation is performed through centroid computation, while the Integral Non-Linearity (INL) is typically critical. However, the INL can be compensated once the TDC conversion characteristic is known.

In case of many TDCs, the different INLs are averaged between them due to the centroid computation, which averages the TDC conversion results. Thus, the INL is reduced compared to that obtained from the single TDC. Typically, DNL and INL lower than 1 LSB peak are adequate to guarantee the required accuracy. The conversion time requirements can be relaxed, even up to a few hundreds of ns, since during the 10 ns FSR, SPADs can only convert one photon due to their dead time, which corresponds to detecting at most one photon per laser pulse (i.e., one photon every 1 μs).

Table 4 summarizes the required performance of the SPAD array.

## 4. Candidate Architectures

In this section, we propose and discuss some architectures of the SPAD TOF array to better match specifics and required performance, considering a 160 nm CMOS technology that allows to reach good SPAD performance in terms of PDP and timing jitter [5,29].

The most demanding requirement is the capability of achieving a 10 mm TOF repetition accuracy with very high ambient background light (up to 3 klx), which must be suppressed without losing signal photons. Photon losses can be due either to optical limitations (i.e., those caused by PDP and FF) or to excessive count-rate (causing either SPAD or TDC saturation). Since the PDP does not depend on the architecture, the main focus should be aimed at maximizing FF, in order to minimize the optical losses. Instead, concerning count-rate saturation, the TOF array should include as many SPADs as possible, to distribute the incoming photons (background and signal) across a larger number of detectors. Furthermore, it is necessary to minimize the SPAD dead time, which causes photon loss, thus reducing SPAD saturation.As already discussed in Section 3.1, a 14 µm diameter SPAD allows detecting on average 1 signal photon and 0.6 ambient photons hitting each SPAD in each laser pulse. However, by reducing the SPAD diameter below 14 µm, the number of SPADs per unit area can increase. The architectures analyzed in this section will consider 10 μm diameter SPADs, since smaller detectors would have worse performance and lower FF [25,26,27]. In this condition, by applying Equations (4) and (5), we obtain, respectively, 0.52 signal photons and 0.32 ambient photons hitting each 10 μm SPAD per pulse: therefore, in the worst case at the longer distance where the 0.32 ambient photons are hitting the SPAD before the laser pulse. As the SPAD is blind after being hit by an ambient photon, the signal photon is lost with a probability of 32%.

The last cause of photon loss is the TDC conversion dead time. An architecture with one TDC per SPAD would give no conversion loss, at the expense of low FF and huge amount of TOF data to process (mostly related to background photons). Instead, a shared TDCs architecture would simplify the chip and reduce the computational effort but at the expense of higher photon losses caused by saturation of the TDC conversion rate. 

For these reasons, these two architectures (i.e., “one TDC per SPAD” and “TDCs shared among multiple SPADs”) will be discussed in the following sections to find the best solution that minimizes signal loss while minimizing the number of required SPADs and TDCs.

### 4.1. One TDC per SPAD

**Figure 5:**
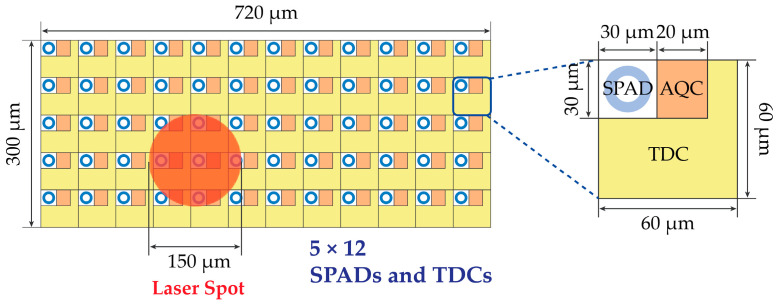
Example of SPAD array with one TDC integrated in each pixel.

The best configuration in terms of maximum acquired data is to have one front-end circuit (e.g., the Active Quenching Circuit, AQC) and one TDC per each SPAD, so that every detected photon is recorded with its TOF. Figure 5 shows a reasonable sizing of the required silicon area for a 150 µm diameter laser spot.

**Figure 6:**
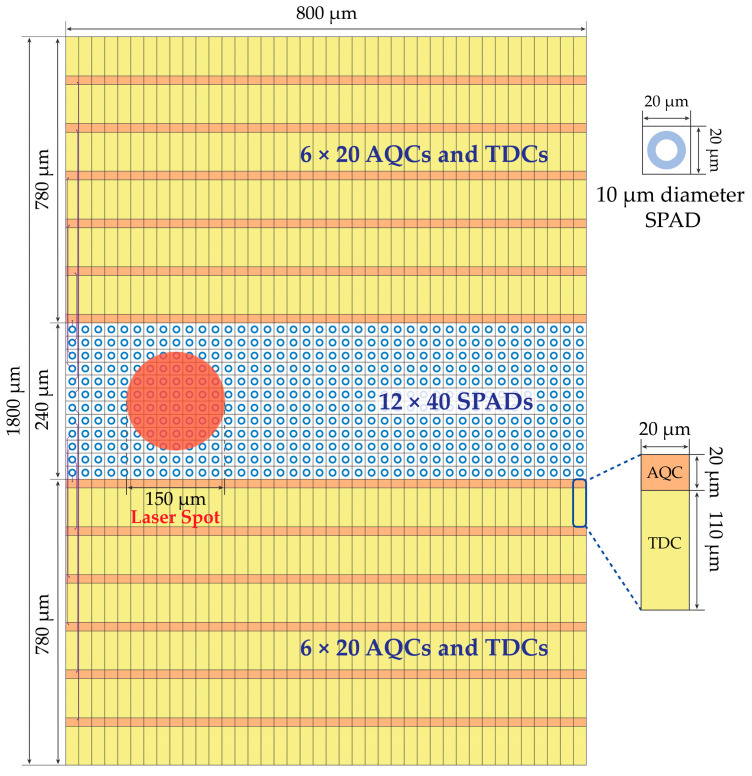
Example of SPAD array with one TDC per SPAD, placed outside the detection area.

In Section 2, we estimated that at least 7.6 signal photons per laser pulse (i.e., 3800 photons considering 500 repetitions) must be detected across the array for achieving the desired 10 mm repetition accuracy (without considering the detrimental effects of background light). Unfortunately, since the 150 μm diameter spot size would cover only about 4–6 pixels, it would be impossible to acquire enough photons to match such a requirement. Since the SPAD array is rectangular (see Section 3.1), another possible configuration is to place all TDCs outside the SPAD array area, as shown in Figure 6, to maximize FF without having electronics among SPADs. With a pitch of 20 µm as shown in Figure 6, about 38 SPADs out of 480 would receive light from the 150 µm diameter laser spot. Considering the Poisson statistics and the detrimental effect of pile-up, this solution allows achieving the target requirement, but it is also quite unpractical, since it requires 480 TDCs instead of 192 TDCs, to manage the full 12 × 40 pixels covering a 0.24 mm × 0.8 mm active area. The total chip area would exceed the target 2 mm × 2 mm, and the data to be read- out, stored, and processed would become unpractical to manage. Therefore, with so many SPADs, each TDCs should be shared among an ensemble of SPAD (defined as a “cell” in the followings).

### 4.2. TDC Shared among Multiple SPADs

When one TDC is shared among multiple SPAD, a common way not to lose important information is to trigger the shared TDC only when multiple SPADs simultaneously trigger (i.e., photons coincidence event). In order to optimize array performance, it is crucial to properly define the number of coincidences required.

**Figure 7:**
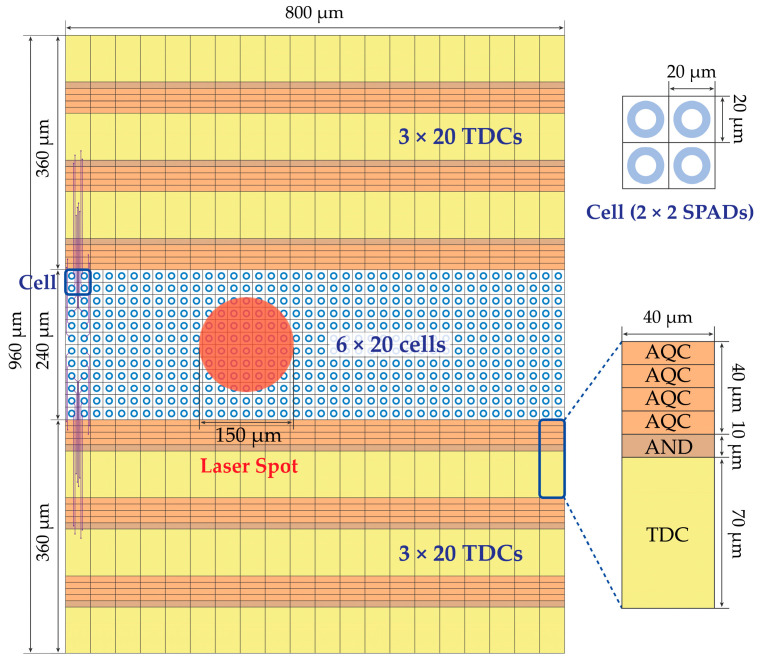
Example of multi-photon SPAD array (**left**), with one TDC shared among a low number (e.g., 2 × 2) of SPADs (**right**); the TDC is triggered by a digital combination (e.g., and AND gate) of the AQCs’ outputs.

**Figure 8:**
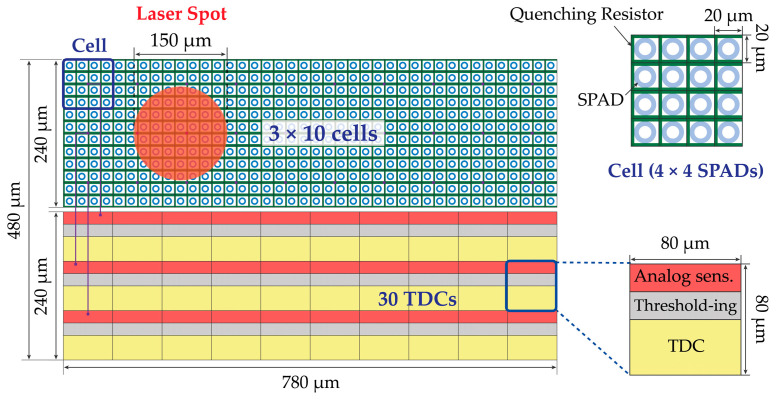
Example of multi-photon SPAD array (**left**), with TDCs shared among a large number (e.g., 4 × 4) of SPADs; an analog sensing block can sum the currents of each SPAD so to trigger the TDC only when a defined threshold is crossed (**right**).

Two different approaches are analyzed in the following sections, which are more convenient when either a few (e.g., up to 3 × 3) or many (e.g., more than 4 × 4) SPADs are grouped within each cell. The former is schematized in Figure 7, in which 2 × 2 SPADs share one TDC; the latter is shown in Figure 8, based on TDCs shared among 4 × 4 SPADs groups. The TDC gets triggered only when a given number of SPADs simultaneously ignite. In the former approach, the threshold can be implemented through a combinatorial logic (e.g., an “AND” gate) [11,12,13,14,15,16,17], while in the latter, the number of digital combinations would be too high to be manageable through logic gates, thus preferring an analog approach with an analog discriminator [32,33]. For example, each triggered SPAD may generate a short current pulse and the sum of all currents in a cell be compared to an analog threshold, within a desired coincidence window. As an additional advantage, this analog approach allows runtime threshold adjustment; on the other hand, it is more sensitive to crosstalk among pixels and disturbances.

All these architectures have a main drawback: the larger the number of SPADs sharing the same TDC, the higher the information loss, since photons detected by many different SPADs are converted into one single TOF data, thus providing only one event in the timing histogram. Indeed, even though about 50 SPADs of the array would be covered by the 150 μm laser spot diameter, sharing one TDC every four SPADs (e.g., a 2 × 2 cell) would limit the maximum number of conversions to less than 13 (50/4) per laser pulse. This result is close to the lower limit to reach the target 10 mm repeat accuracy (which requires at least 7.6 signal photons per laser pulse) and is further degraded because of other signal losses (e.g., count rate saturation of each SPAD in the cell).

For this reason, the standard approach of many SPADs sharing one TDC will not be further taken into consideration. Instead, we propose a novel architecture that allows maximizing photon detections and TOF measurements, given the available TDCs, so to reach the required accuracy.

## 5. Laser Spot Tracking

The main purpose of the optimization is to skip the SPADs not covered by the laser spot, tracking the laser spot [16], thus selecting a smart Region of Interest (ROI) and then minimizing the number of TDCs while still being sensitive to all useful signal photons within the spot by means of a smart TDC sharing [15,17,34].

The laser spot tracking and smart ROI selection exploits an a priori estimation to assess whether each SPAD falls within the laser spot or not, thus neglecting any triggering of off-spot SPADs. To ascertain if a SPAD is within the laser spot, we propose to count the total number of SPAD ignitions when the laser echo signal is expected by running a digital counter upwards during the 10 ns of the FSR time duration and then to subtract the number of background events by running the same counter downwards, during another 10 ns time slot when no laser photons are expected.

**Figure 9:**
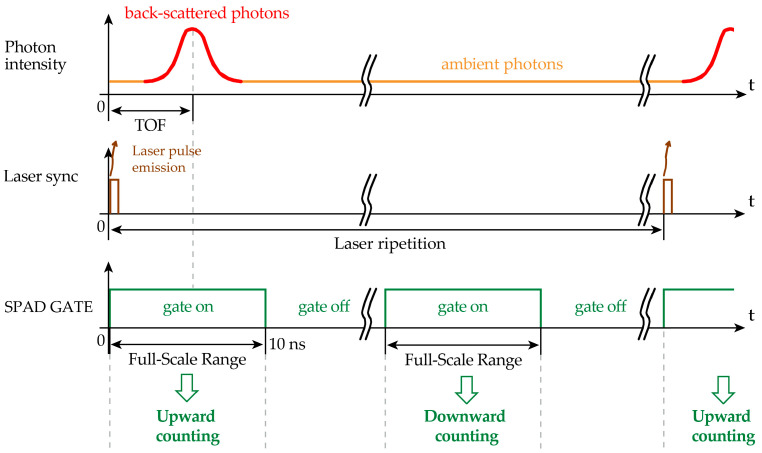
Timing diagram of the signals to properly count (**upwards**) signal and background photons and to subtract (**downwards**) only ambient background photons, during one laser period.

**Figure 10:**
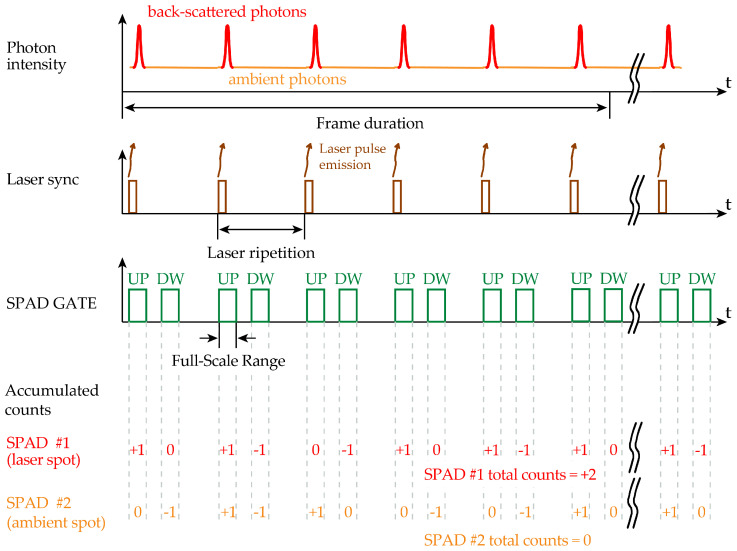
Timing diagram of the signals to properly count (**upwards**) signal and background photons and to subtract (**downwards**) only background photons, during one frame (i.e., 500 laser pulses). Two SPADs are considered, SPAD #1 inside and SPAD #2 outside the laser spot, thus accumulating a total positive count and an average nil count, respectively.

Figure 9 shows the proper timings of the control signals within one laser pulse period, while Figure 10 shows the repetition of 500 laser shots, which represent a frame and two exemplifying cases of a generic SPAD #1 within the laser spot and another generic SPAD #2 outside the laser spot. The counter of SPAD #1 will accumulate on average a positive result, while the one of SPAD #2 will result in no counts on average. Note that within each counter enabling window (either Up or Down), the accumulated counts can increment (+1) or decrement (−1) just by 1 event, at the most: the reason being that each SPAD can be triggered only once within each enabling window (SPAD GATE).

Since 500 laser shots are allowed within the specified response time and at the laser repetition frequency, 500 enabling windows in both upward and downward counting are typically enough to clearly distinguish which SPAD is within or outside the laser spot.

Therefore, the up/down counter could accumulate up to 500 increments, in case of very strong laser signal intensity with no background light; thus, a 10-bit (9-bit plus 1-sign bit) up/down counter is required.

Depending on whether the count accumulated by each counter at the end of each frame exceeds a user-selectable threshold, the output of the corresponding SPAD front-end is either allowed or not to trigger a TDC. In this way, the conversion rate and the number of useless TOF measurements are drastically reduced. Note that all SPADs are kept enabled in any case, so that their counters can be always operative for determining the spot position for the following frame, in case the laser spot changes position.

**Figure 11:**
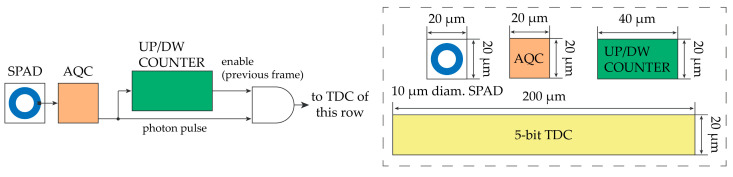
Possible hardware implementation of the laser spot tracking (**left**) and tentative dimensions of SPADs, AQCs, counters, and shared TDCs in a 160 nm CMOS technology (**right**).

Figure 11 shows the block diagram of the smart ROI selection circuitry and the tentative size of each block. Each SPAD is sensed by the corresponding AQC, which provides a digital pulse every time the SPAD is ignited by a photon (or a dark count). A similar more complete implementation is shown in [16], which basically uses a counter in up and down counting to identify the laser spot position. However, the identification of the spot is performed off-chip after evaluating the up and down counts using external processing. Instead, in this proposed implementation, the ROI selection is performed on-chip and it depends by the user-selectable threshold.

**Figure 12:**
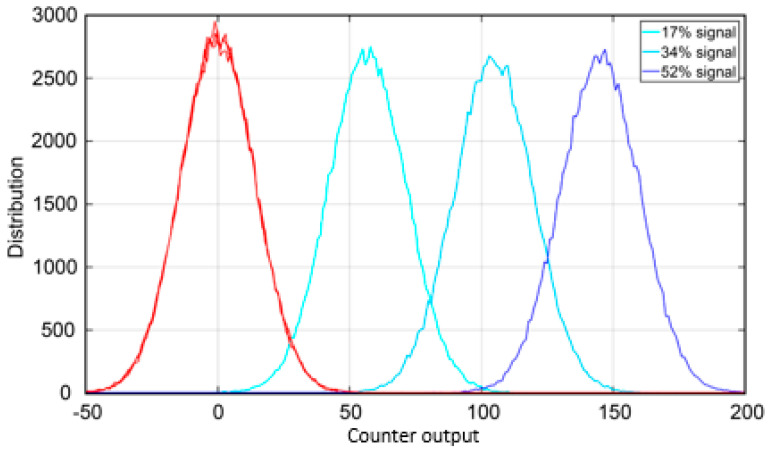
Up/down counter’s output after 500 laser shots, achieved with 10^6^ Monte Carlo simulations in the worst-case conditions (lowest laser echo signal). The red curve represents SPADs not covered by the laser spot (resulting in 0 counts on average), whereas the blue and light blue curves represent SPADs covered by the laser spot, respectively with 52%, 34%, or 17% probability to detect a photon per SPAD per each laser shot.

Figure 12 shows a Monte Carlo simulation of the statistical distribution of the counts accumulated by the up/down counters, in different illumination conditions. With background photons only (red curve), the counters reach a distribution centered around zero on average. In contrast, SPADs illuminated by the laser spot (blue curves) give a counting distribution that moves toward positive values. We considered 52%, 34%, or 17% probability to detect a photon for each laser shot, i.e., the actual situation to have on average 0.52 photons per SPAD per laser shot and also two pessimistic situations (0.34 and 0.17 photons per SPAD per laser shot) of signal below the expected minimum. Due to the random nature of the detection process, the distribution has a not-nil width; therefore, it is not possible to check if the counter has a zero or not-nil value. Instead, it is necessary to set a threshold. For the 52% case, the two distributions do not overlap: therefore, it is easy to select an optimal threshold within the 50–100 range (see Figure 12).

For example, setting the fixed threshold to 64 counts could be a smart choice, since it is in the middle of the non-overlapping region and it corresponds to the setting of the 7th bit of the counter, thus easing the threshold crossing check. Nevertheless, in this case, also a threshold at 100 would be still fine. However, setting a fixed threshold can introduce equivocation errors in case the signal intensity is reduced much below the 52% signal probability, possibly causing an SPAD within the laser spot to be erroneously turned off.

This is what happens in Figure 12 when a threshold at 100 is used also for the 34% probability case: about half of the SPADs within the laser spot would be considered to be outside, instead. In order to operate even down to 17% laser detection probability, a threshold at 30 could be a reasonable trade-off to discriminate in-spot and off-spot SPADs. Note that the 17% signal probability is unrealistic for SPADs not located on the edge of the laser spot: a threshold at 64 counts would cause the SPADs actually within the spot to be anyhow unable to trigger the TDC in about 50% of the cases.

**Figure 13:**
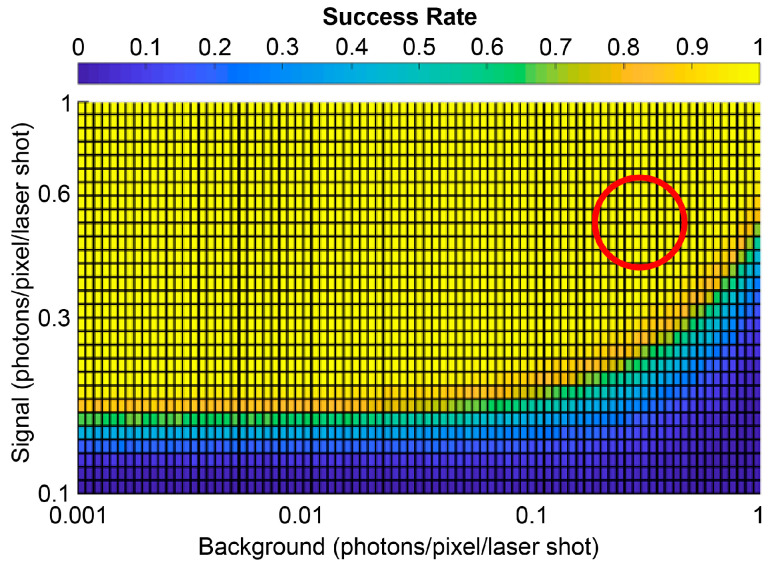
Monte Carlo simulation showing the success ratio in considering an SPAD within the spot as a function of the signal detection rate and the background detection rate. The red circle shows the region with 0.52 signal photons and 0.32 background photons per laser shot per SPAD.

Figure 13 shows the results of Monte Carlo simulations computing the success rate; i.e., the probability of performing a correct SPAD evaluation, causing neither SPADs actually inside the spot to be discarded nor SPADs outside the spot to be erroneously enabled to trigger the TDC. The threshold was kept constant at 64 photons, and the signal detection rate spans from 0.1 up to 1 photon per SPAD and per laser shot, while background from 0.001 up to 1 photon per SPAD per FSR. Note that with a 0.1 signal detection rate, the success probability is almost 0% even with very low background, because on average, 50 signal photons are detected (0.1 times the total 500 laser shots per each frame), which are not enough to exceed the threshold.

**Figure 14:**
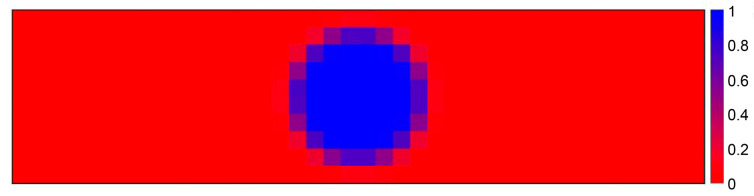
Monte Carlo simulation showing the probability to identify each SPAD of the 10 × 40 SPAD array as being inside the laser spot, when a 130 µm FWHM Gaussian spot hits the array, in the worst signal to background ratio conditions.

In Figure 14, we consider an SPAD array consisting of 10 rows by 40 columns, i.e., 400 SPADs, and we illuminate the array with a Gaussian laser spot (130 µm FWHM) in the worst-case conditions, i.e., with the highest possible background level and the weakest possible signal intensity. The Monte Carlo simulation gives the probability to identify each SPAD of the array as being within the laser spot: a high probability indicates that the SPAD is within the spot; a low probability indicates that the SPAD is outside it.

As can be seen in Figure 14, the proposed method is actually able to correctly identify if the SPAD is under the photons of the laser spot. All SPADs outside the laser spot have 0% probability of triggering the corresponding TDCs, whereas the central pixels of the laser spot have a 100% probability to trigger them. We also performed simulations in dynamic conditions, with the laser spot moving across the SPAD array with a speed of 1 column every 500 µs. The circuitry is able to track the laser spot with no uncertainty.

## 6. Smart TDC Sharing

Thanks to the smart ROI selection, only the SPADs hit by the laser spot are enabled to trigger the TDCs through the AND gate shown in Figure 11 to perform TOF measurements. Thus, in order to avoid signal loss, neighboring SPADs that may be covered by the laser spot should be connected to different TDCs. Therefore, we propose a second smart architecture, which was aimed at smart TDC sharing [15,16,17,34] performed across SPADs belonging to the same row (e.g., the side where the spot is expected to drift along).

The number of TDCs per row is chosen to reduce area consumption and to best accommodate the laser spot, keeping some margin to comply with possibly variable diameter laser spots, e.g., depending on the optical setup and the object distance. Each row has the same number of available TDCs, even if the rows at the center of the laser spot show the higher number of illuminated columns (hence of TDCs), while those at the edges of the laser spot may have only one column illuminated due to the circular shape of the spot.

Each channel will feed one TDC (e.g., from TDC A to TDC H), but only if allowed to (by means of the smart ROI selection discussed in Section 5). Only those SPADs that resulted within the laser spot in the previous frame are enabled to trigger the corresponding horizontal line feeding the TDC. If more than one SPAD is enabled along one TDC line (e.g., SPAD in rows 1 and 9 and 25, all corresponding to channel A), there will be no conflict as only the first ignition event will trigger the TDC. Thus, in a 12 × 40 array, by using N = 8, only eight TDC per each one of the 12 rows are needed, corresponding to a total of 96 TDCs instead of 480 TDCs, i.e., just 20% of them.

**Figure 15:**
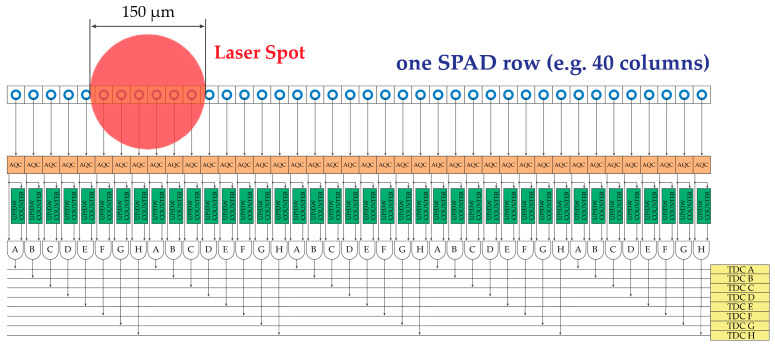
Example of one row of the SPAD array: by grouping columns together in N channels (e.g., N = 8, from A to H), only N TDCs are required per each row whatever number of actual columns.

Given a spot diameter of 150 µm and an SPAD pitch of 20 µm, the maximum number of adjacent columns (e.g., long side of array) within the laser spot will never exceed 7. Therefore, a reasonable trade-off between minimizing the number of TDCs and maximizing the number of SPADs enabled to ignite a TDC is to use N = 8. Figure 15 shows the concept applied to a full row of the SPAD array, e.g., with 40 columns. Instead of using several TDCs equal to the columns of the SPAD array (i.e., one TDC per column), we propose to group columns (i.e., SPADs and AQCs) in five groups of N = 8 channels.

## 7. TOF Chip Floor-Planning

**Figure 16:**
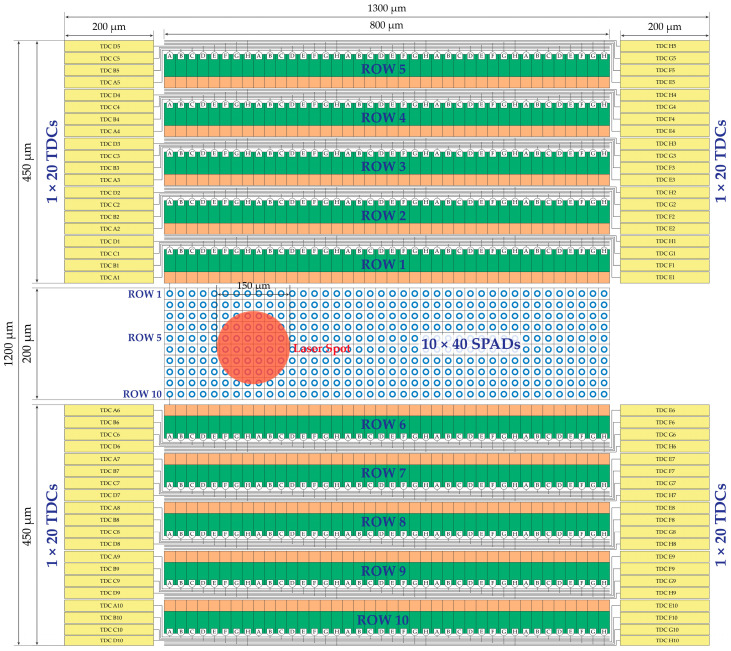
SPAD and TDC arrays blocks layout of the TOF chip; note that the global electronics, storage memory, data processing, and read out circuitry are not shown.

Figure 16 shows an example of the full architecture of SPAD TOF array, based on 10 × 40 SPADs and four banks of 20 TDCs each (organized in five groups of four TDCs) for a total of 80 TDCs, employing reasonable sizing for a 160 nm CMOS technology. The number of TDC groups is equal to the number of rows, while the number of TDCs per each group is equal to the number N of elements within each AQCs subset (dived in two equal parts in the right and left banks). Since the number of columns is 40, while N = 8, the number of TDCs is only 1/5 of the number of SPADs. Note that increasing the number of columns would increase the SPAD and AQC and counter array but not the number of TDCs.

To optimize area occupation and guarantee symmetry, all groups of eight TDCs per each row have been split in half on the left-hand side and half on the right-hand side of the AQC arrays. Note that the TDCs have been labeled accordingly: for example, “TDC E4” is the TDC of the fourth row, being driven by one (or more) of the SPADs of columns E, i.e., column 5, 13, 21, 29, and 37.

With this type of floorplan, timing skew can be presented from different SPAD to TDC. However, considering post-layout simulations on a 160 nm CMOS technology, the timing skew can be negligible by comparison with the target TDC resolution (TDC LSB in the order of 100 ps). Thus, the required accuracy will not be compromised.

The overall arrays of SPADs, AQCs, and counters, and finally TDCs occupy an area of about 1200 µm × 1300 µm. Given the specifications of a total chip area of 2 mm × 2 mm, the remaining area of 800 µm × 700 µm is available for the remaining electronics of the TOF SPAD array, including the memory banks for storing the TDC results, the data processing to compute the averaged TOF, and the I/O pads.

## 8. Conclusions

After considering two possible candidate architectures for the TOF SPAD array, one based on one TDC per each SPAD and one based on a shared TDCs approach, we showed that none of these solutions allow guaranteeing the project requirements. For these reasons, we proposed an optimized solution in which each TDC is shared among few SPADs through a smart ROI selection and TDC sharing, so that almost no information is lost in the TDC conversion, and at the same time, the signal-to-background ratio is maximized by disabling detectors not detecting photons from the active illumination.

Compared to other solutions, such as the ones based on SPAD threshold (i.e., in which the TDC is only triggered after a selectable number of concurrent detections), this architecture allows maximizing the number of TDC conversions, thus allowing to reach the best measurement precision by means of centroid computation.

## Figures and Tables

**Table 1:** State-of-the-art of SPAD imagers for TOF-LiDAR.

Ref.	Type	Pixel Number	IC Area (mm^2^)	Pixel Size (µm^2^)	Fill-Factor (%)	Resolution (mm)	Range (m)
[5]	Photon-timing (one TDC per SPAD)	32 × 32	4.8 × 4.8	150 × 150	3.14	58.5	60
[6]	Photon-timing (one TDC per SPAD)	32 × 32	1.6 × 1.6	50 × 50	2.3	17.9	18
[7]	Photon-timing (one TDC per SPAD)	60 × 1	9.3 × 2	150 × 150	52	30	38.4
[8]	Time-gated photon-counting	64 × 32	9.6 × 4.8	150 × 150	3.14	3	2.4
[9]	Time-gated photon-counting	128 × 128	3.2 × 3.2	25 × 25	4.5	30	1.44
[10]	Time-gated photon-counting	256 × 256	N.D.	36 × 43	11	3000	1500
[11]	Multi-photon (shared TDC)	64 × 64	3.8 × 3.8	30 × 15	25	3.75	200
[12]	Multi-photon (shared TDC)	32 × 32	4.2 × 4.6	100 × 100	9.6	11.3	30.7
[13]	Multi-photon (shared TDC)	340 × 96	1.6 × 0.15	25 × 25	70	3.12	1.28
[14]	Multi-photon (shared TDC)	8 × 16	9.8 × 1.6	40 × 16	36	0.98	3.93
[15]	Multi-photon (shared TDC)	144 × 252	21.6 × 10.2	28.5 × 28.5	28	1.4	50
[16]	Multi-photon (shared TDC)	50 × 40	3.3 × 2.9	38.5 × 33.5	4.8–15.3	6.02	7.5
[17]	Multi-photon (shared TDC)	256 × 8	11.6 × 2.1	41.6 × 41.6	35	2.92	35
[18]	Multi-photon (shared TDC)	256 × 256	0.25 × 0.8 (Top tier)	19.8 × 19.8	31.3	9	150–430
[19]	Multi-photon (shared TDC)	256 × 256	2.46 × 2.46 (Top tier)	9.2 × 9.2	51	5.25	50

**Table 2:** Specifications and performance requirements for the single-point TOF sensor.

	Parameter	Value
Sensing performance	Range	>1 m
	Measurement precision	<±10 mm (±3 σ value)
	Frame time	0.5 ms
Ambient and target	Ambient light illuminance (halogen lamp)	3000 lx
	Ambient reflectivity	50 %
	Target reflectivity	3 %
Laser active illumination	Wavelength	660 nm
	Light peak power	100 mW
	Pulse width (FWHM, Full Width at Half-Maximum)	<3 ns
	Repetition rate	1 MHz
	Laser spot diameter at detector level	150 µm
Optics	Objective diameter	10.7 mm
	Numerical aperture	0.357
	Filter transmissivity	85%
	Lenses transmissivity	85%
Sensor chip	Total area	<2.0 × 2.0 mm^2^

**Table 3:** Minimum number of photons to be detected and expected photon rates.

Item	Value
Minimum number of photons to be detected	≈7.6 ph (per laser pulse)
Signal detection rate	≈3.4 × 10^4^ ph/mm^2^ (per laser pulse)
Background detection rate	≈2 × 10^12^ ph/s/mm^2^

**Table 4:** Required performance and geometrical parameters of the SPAD array.

	Parameter	Value	Comment
Geometrical parameters	Array shape	Rectangular	Considering the 1D displacement
	SPAD area	<150 µm^2^	SPAD diameter <14 µm, considering round-shaped active area
SPAD performance	DCR	<1 Mcps	Not critical, compared to ambient background
	PDP	≈20% (at 660 nm) 5% (at 905 nm)	Low PDP is not so critical since it allows to reduce the count-rate saturation
	Timing jitter	<100 ps FWHM	Negligible, time jitter dominated by the laser
	Dead time	>10 ns	Limiting afterpulsing, but also the SPAD count rate to one photon per laser pulse
TDC performance	LSB	<100 ps	To be negligible compared to the laser width
	FSR	≈10 ns	For a maximum distance of about 1 m
	Linearity	<1 LSB	DNL and INL can be reduced by means of the centroid computation
	Conversion Time	<1 µs	One conversion (one photon) for laser pulse
	TOF samples	500	Defined by the response and acquisition time

## Data Availability

Not Applicable.
